# A Case of Immune Checkpoint Inhibitor Refractory Colitis Treated with Mycophenolate and High-dose Steroids

**DOI:** 10.7759/cureus.6392

**Published:** 2019-12-16

**Authors:** Daniel Alcantar, Layth Al-Jaashaami, Fanny Giron

**Affiliations:** 1 Internal Medicine, MacNeal Hospital, Berwyn, USA; 2 Gastroenterology, Banner University Medical Center, Phoenix, USA

**Keywords:** colitis, melanoma, colonoscopy, mycophenolate, immune checkpoint inhibitors

## Abstract

Immune checkpoint inhibitors (ICPI) are a class of chemotherapy agents that have emerged as a front-line treatment option for multiple cancers. Nivolumab is an ICPI agent commonly used to treat metastatic melanoma. Although promising, the adverse reaction of this class is broad and per reports, the incidence of colitis is <6%.

We present the case of a 71-year-old male with a history of metastatic melanoma who was being treated with nivolumab. The patient was two weeks into his treatment regimen when he began complaining of multiple loose, bloody stools. Because of these symptoms, his nivolumab was discontinued. However, despite discontinuation, his symptoms persisted and the patient underwent a colonoscopy. He was found to have diffuse inflammation of the colon and was diagnosed with nivolumab-induced colitis. Subsequently, the patient underwent multiple treatments, including high-dose steroids, infliximab, and vedolizumab (Entyvio), with no resolution of symptoms. After several months of failed treatment, the patient was readmitted to the hospital for refractory colitis. He was started on high-dose steroids and underwent a repeat colonoscopy, which again showed diffuse colitis. Because of the previously failed treatment options, mycophenolate, an immunosuppressant, was initiated in combination with his steroids. After three days of high-dose steroids and mycophenolate, the patient's symptoms resolved, with no subsequent apparent symptoms of colitis.

We present a case of nivolumab-induced colitis, refractory to multiple immunosuppressive medications, which was successfully treated with mycophenolate and high-dose steroids.

## Introduction

Cancer is a major public health problem worldwide and is the second leading cause of death in the United States. Fortunately, there has been a decline in cancer death rates over the last two decades, with an overall drop of 26%, resulting in 2.4 million fewer cancer deaths during this time period [1]. Immune checkpoint inhibitors (ICPIs) have made tremendous strides in recent years and have emerged as a front-line treatment option for multiple cancers such as metastatic melanoma, non-small cell lung cancer (NSCLC), renal cell carcinoma (RCC), and bladder or urothelial cancer [2]. Nivolumab is an agent in the class of ICPIs. It is a protein-1 (PD-1)/protein-1 ligand (PD-L1) inhibitor that targets T cells at a later stage of the immune response within the tumor and peripheral tissues. PD-1 is a receptor found on monocytes, T cells, B cells, dendritic cells, and tumor-infiltrating lymphocytes. PD-1 binds to PD-L1 (which is overexpressed in tumor cells and antigen-presenting cells), suppressing T-cell receptor signaling responses [3].

Unfortunately, ICPIs have been associated with serious immune-related adverse events due to the over-activation of the immune system. These adverse events can affect any organ but most commonly affect the gastrointestinal tract, liver, endocrine glands, and skin. Immune checkpoint inhibitor-associated colitis can be challenging to diagnose, as there are other potential causes of diarrhea and the onset and severity of immune-related colitis is variable (typically within weeks to a couple of months) [3]. The Common Terminology Criteria for Adverse Events (CTCAE) are a set of criteria used to classify the adverse effects of drugs in clinical trials including cancer therapy. Based on the CTCAE grading (severity) scale, the treatment options may vary from the symptomatic treatment of diarrhea with loperamide and electrolyte repletion to the initiation of immunosuppressive agents [4]. In the case of refractory colitis, multiple studies have shown a response to infliximab, mycophenolate, cyclosporine, and Entyvio [5-8]. We present a case of refractory colitis to both infliximab and Entyvio, treated with a high-dose steroid (methylprednisolone) and mycophenolate.

## Case presentation

We present the case of a 72-year-old male with a significant medical history of prostate cancer (in remission) and a history of metastatic melanoma (status post right upper lobe resection) who presented to the emergency department secondary to multiple bouts of bright red blood per rectum for several months. Per patient, he was receiving adjuvant chemotherapy nivolumab six months prior to the admission, and it was discontinued because he began experiencing multiple bouts of bloody diarrhea daily and was diagnosed with nivolumab-induced colitis.

The patient, at that time, was prescribed high-dose steroids in combination with mesalamine but the treatment was unsuccessful. He underwent a colonoscopy that showed sigmoid colitis and procto-colitis. Because of this, the patient was restarted on steroids and was initiated on infliximab infusions. The patient again received a total of six weeks of steroids as well as two infliximab infusions; unfortunately, he continued to complain of 15-30 bloody bowel movements daily. He underwent a repeat colonoscopy, which again revealed procto-colitis. Due to these findings, as well as the continuation of symptoms, the infliximab was discontinued and he was started on Entyvio. Despite two infusion doses of Entyvio and a steroid taper, he continued to be symptomatic, complaining of 15-30 bloody bowel movements daily. Owing to the continuation of symptoms, the patient was readmitted to the hospital and high-dose steroid (methylprednisolone 60 mg intravenous (IV) three times a day (TID)) were initiated.

**Figure 1:**
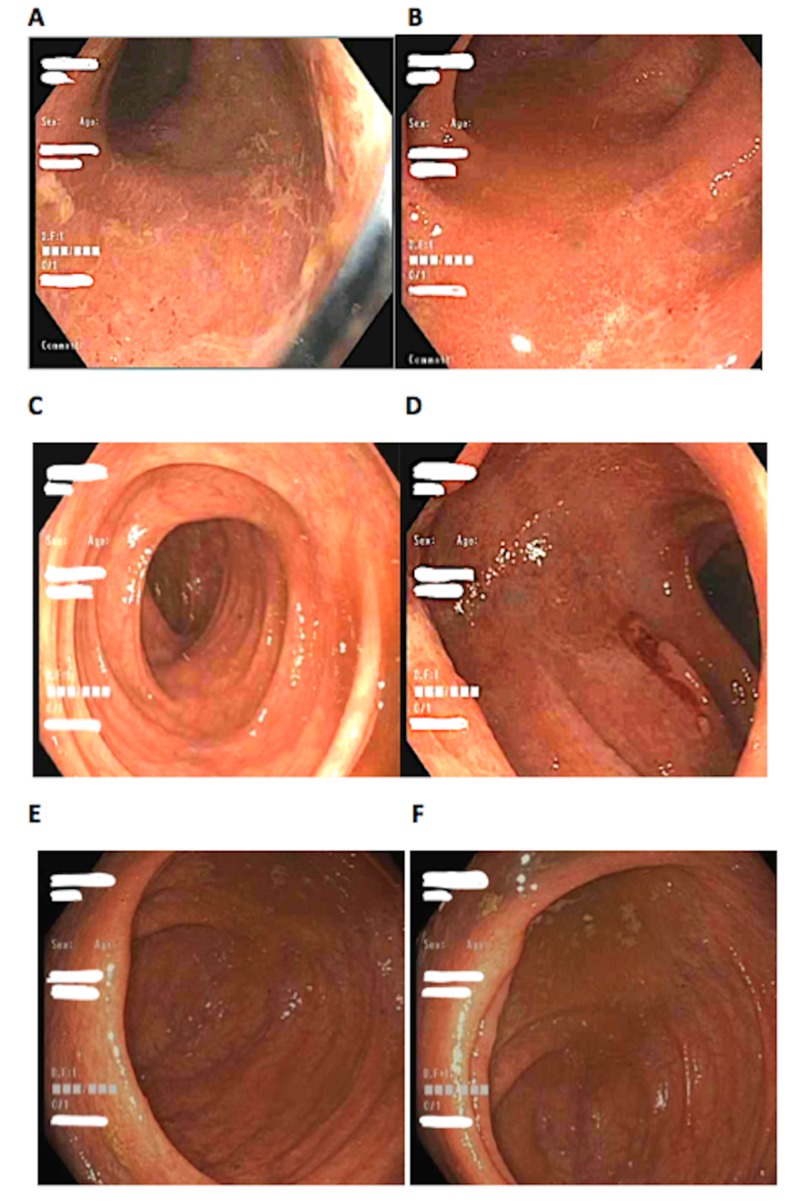
Image A-F showing diffuse patchy erythema from the rectum to the ileocecal valve. Image A, rectum. Image B, sigmoid colon. Image C, ascending colon. Image D, transverse colon. Image E, cecum. Image F, ileocecal valve

The following day, the patient underwent a colonoscopy and was found to have diffuse patchy inflammation from the rectum to the cecum (Figure 1) and biopsies were obtained, which revealed severe active chronic colitis in the right colon, sigmoid, and rectum. Post-colonoscopy, the patient was initiated on mycophenolate 1000 mg twice per day (BID) in conjunction with methylprednisolone. After three days of treatment, the patient’s symptoms had resolved, and he was inevitably discharged home with a prednisone taper as well as mycophenolate 1000 mg BID.

Five weeks after discharge, the patient followed up in the clinic and was successfully tapered off prednisone while continuing mycophenolate 1000 mg BID. At that time, he continued to deny any further episodes of bloody diarrhea and endorsed having soft bowel movements daily. Because of this, the mycophenolate was tapered to 750 mg BID and was started on Lialda 4.8 g daily (QD). Ten weeks after discharge, he continued to deny any additional flares and was doing well with the current regimen of mycophenolate 750 mg BID and Lialda 4.8 mg QD. The decision was made to continue mycophenolate taper, reducing the dose to 500 mg BID and Lialda was continued at the same dose. The patient was asked to follow up in five weeks.

## Discussion

Colitis is a well-known side effect of the ICPIs. In a recent comprehensive review, it was found that patients receiving anti-PD-1 inhibitors (e.g. nivolumab) have a risk of 1.3%-2.9%. Other classes of ICPIs such as anti-CTLA-4 and anti-PD-L1 showed a risk of 7.0%-11.6% and 0.7%-19.7% [4]. When comparing anti-PD-1 inhibitors to the other class of ICPIs, the frequency and severity of colitis seem to be lower. The severity of nivolumab-induced colitis was very low, for which it was reported as grade 3-4 diarrhea in 1.0%-2.0% of patients [9]. Furthermore, colitis is typically more frequent and severe with combination immunotherapy [3].

With regards to the grading severity of immune-related toxicities, Common Terminology Criteria for Adverse Events (CTCAE) version 5.0 is used (Table 1) [10]. Depending on the grade of the toxicity, it provides further guidance into treatment recommendations.

**Table 1:** Common Terminology Criteria for Adverse Events (CTCAE) version 5.0 ADL = activities of daily living Adapted from the Cancer Therapy Evaluation Program, National Cancer Institute Common Terminology Criteria for Adverse Events v5.0 Program, Common Terminology Criteria for Adverse Events v5.0 https://ctep.cancer.gov/protocolDevelopment/electronic_applications/docs/CTCAE_v5_Quick_Reference_8.5x11.pdf

Grade	1	2	3	4	5
Diarrhea/colitis	Increase of <4 stools per day over baseline (or mild increase in ostomy output compared with baseline) without colitis symptoms	Increase of 4-6 stools per day over baseline (or moderate increase in ostomy output compared with baseline) and/or colitis symptoms limiting instrumental ADLS	Baseline (or severe increase in ostomy output compared to baseline), colitis symptoms interfering with ADLs; incontinence; hospitalization indicated; limiting self-care ADL	Life-threatening consequences (e.g., perforation, hemodynamic instability); urgent intervention indicated	Death

The work-up for ICPI-associated colitis is performed for patients with grade 2 toxicities or above. The common laboratory tests ordered include complete blood count (CBC), erythrocyte sedimentation rate (ESR), and C-reactive protein (CRP) [3]. Additional workup or laboratory tests, such as fecal lactoferrin or calprotectin, if elevated, may also help point to an inflammatory cause [10]. Ultimately, endoscopy (i.e. colonoscopy or flexible sigmoidoscopy) with biopsies is the recommended choice for diagnosis and is recommended for patients with persistent grade 2 colitis and patients with grade 3-4. Typical endoscopic findings include loss of vascular pattern, exudates, granularity, friability, and ulcerations [3].

Treatment requires early recognition and is based on the severity of colitis determined by the CTCAE. In a recent comprehensive review, the management for grade 1 colitis was to continue with ICPI therapy with symptomatic treatment of diarrhea with loperamide and electrolyte repletion. For grade 2, ICPI are withheld and oral corticosteroids at 0.5-1.0 mg/kg are initiated with a taper over one to two months. For grade 3 or 4 toxicities, ICPIs are permanently discontinued, patients are typically hospitalized, and systemic corticosteroids are initiated at 1.0-2.0 mg/kg per day. If no response to high-dose steroids, or if relapse requires an increase in steroid dosing during the tapering period, such cases are considered refractory [4]. In cases of refractory colitis, four different medications were investigated in recent studies, which include: infliximab, mycophenolate, and Entyvio [5-8].

In our present case, our patient was considered grade C toxicity. The patient had received multiple different treatment options for his refractory colitis. He initially began with high-dose steroids, which was unsuccessful. He then underwent the combination of high dose steroids with infliximab injections, for which he had received a total of three doses. This option was also unsuccessful, as symptoms persisted; unfortunately, drug trough levels were not obtained for a possible escalation of medication [4]. The patient then underwent Entyvio infusions. He received a total of two infusions, with again no resolution of symptoms. The Entyvio was then discontinued and mycophenolate was initiated in combination with high-dose steroids (methylprednisolone 60 mg TID). Mir et al. [6] treated refractory colitis patients with the combination of IV steroids and Mycophenolate initially and if refractory to this treatment, they were initiated on infliximab. Interestingly, when comparing our case to the study by Mir et al., our patient was refractory to infliximab initially and symptoms improved with the combination of IV methylprednisolone 60 mg TID and 1000 mg of mycophenolate BID. 

To our knowledge, this is the first case of refractory colitis that was resistant to both infliximab and Entyvio. Given the increase in the use of ICPIs within the past years, it is important for gastroenterologists to familiarize themselves with the possible side effects of these medications as well as consider the various amounts of options that are available in cases of refractory colitis.

## Conclusions

Prompt identification of ICPI-related colitis could be challenging, as there are other potential causes of diarrhea and the timing of onset and severity are variable. Early diagnosis is important in order to prevent complications such as persistent or worsening colitis. In cases of refractory colitis, although there are multiple treatment options to consider, further studies should be performed in order to better identify the best treatment choice for long-term management in patients with ICPI-associated refractory colitis.
